# Competition of dual *SF3B1*^mt^ clones in MDS-RS is associated with distinct RNA mis-splicing in hematopoietic stem cells^∗^

**DOI:** 10.1016/j.bneo.2024.100011

**Published:** 2024-04-12

**Authors:** Pedro Luis Moura, Yasuhito Nannya, Affaf Aliouat, Isabel Juliana Hofman, Teresa Mortera-Blanco, Tetsuichi Yoshizato, Ryunosuke Saiki, Masahiro M. Nakagawa, Maria Creignou, Ann-Charlotte Björklund, Gunilla Walldin, Indira Barbosa, Monika Jansson, Francesca Grasso, Edda M. Elvarsdottir, Petter S. Woll, Sten Eirik W. Jacobsen, Seishi Ogawa, Eva Hellström-Lindberg

**Affiliations:** 1 Center for Hematology and Regenerative Medicine, Department of Medicine Huddinge, Karolinska Institutet, Huddinge, Sweden; 2 Department of Pathology and Tumor Biology, Graduate School of Medicine, Kyoto University, Kyoto, Japan; 3 WPI Institute for the Advanced Study of Human Biology, Kyoto University, Kyoto, Japan; 4 Institute of Medical Science, University of Tokyo, Tokyo, Japan; 5 Hematopoietic Stem Cell Laboratory, Molecular Haematology Unit, Medical Research Council Weatherall Institute of Molecular Medicine, Radcliffe Department of Medicine, University of Oxford, Oxford, United Kingdom; 6 Division of Hematology, Department of Medicine, Karolinska University Hospital, Huddinge, Sweden; 7 Department of Cell and Molecular Biology, Karolinska Institutet, Stockholm, Sweden

**TO THE EDITOR:**

The core spliceosome component *SF3B1* is one of the most frequently mutated genes in myeloid neoplasms (MN), including myelodysplastic syndromes (MDS).^1^^,^^2^ Similarly to other MN mutations, *SF3B1* mutations (*SF3B1*^mt^) also confer a fitness advantage to mutant hematopoietic stem cells (HSCs).^3^^,^^4^ The molecular mechanisms underlying this competitive advantage of *SF3B1*^mt^ cells and how they relate to clinical disease development are not yet understood. However, recent large-scale studies of myeloproliferative neoplasms and acute myeloid leukemia have demonstrated that initiating fitness advantage-granting mutations frequently become clinically relevant only after expansion.^3^, ^4^, ^5^

Although several studies have investigated the impact of different *SF3B1*^mt^, these have been limited to comparing cells from separate patients with *SF3B1*^mt^ and could therefore be confounded by differences in genetic background and clone-extrinsic factors.^6^^,^^7^ Albeit less frequent than expected by single mutation frequencies in myeloid malignancies (supplemental Table 1), the co-occurrence of separate *SF3B1*^mt^ clones within the same patient provides a unique opportunity to investigate this question.

Herein, we pursue a long-term investigation of the clinical profile, fitness ability, and clonal/phylogenetic dynamics of independent *SF3B1*^mt^ clones found in one individual with MDS with ring sideroblasts (MDS-RS) followed up in the clinic over 14 years (Patient 1, *SF3B1*^N626D/K666N^) and a second individual with dual *SF3B1*^mt^ MDS-RS sampled only at diagnosis (Patient 2, *SF3B1*^K700E/K666N^). Through longitudinal analysis of Patient 1, we demonstrate that RNA mis-splicing is prevalent in the HSC, the cell of disease origin;^8^ and that *SF3B1*^K666N^ and *SF3B1*^N626D^ HSC display mutation-specific RNA mis-splicing profiles with distinct functional enrichment results.

Bone marrow (BM) samples from patients with MDS-RS were collected, subjected to myeloid panel sequencing at Karolinska University Hospital and processed according to established protocols.^9^ Control samples were collected from 4 healthy age- and sex-matched normal BM donors. An overview of patients with dual-SF^mt^ (*SF3B1*, *SRSF2*, and *U2AF1*) in the clinical cohorts of Karolinska University Hospital and Kyoto University is provided in supplemental Table 1 and supplemental Figure 1. All material was provided with written informed consent for research use, given in accordance with the Declaration of Helsinki, and the study was approved by the ethics research committees at Karolinska Institutet (2010/427-31/3, 2017/1090-31/4) and Kyoto University (G608).

Patient 1 was investigated at 64 years of age because of mild anemia. Clinical investigation confirmed MDS-RS with hemoglobin at 12.1 g/dL, mean corpuscular volume of 103 fL, >40% RS, and 2 independent *SF3B1* mutations (variant allele frequency [VAF] of 43% *SF3B1*^N626D^ and 4.6% *SF3B1*^K666N^) without any other recurrent driver mutations. The patient remained untreated but was regularly followed up. Over 14 years, both clones inverted in frequency (Figure 1A; *SF3B1*^N626D^: 43%→0% VAF; and *SF3B1*^K666N^: 4.6%→41% VAF), with a slow, consistent improvement of erythroid parameters (supplemental Figure 2) coinciding with a mild increase in megakaryocyte-erythroid progenitor (MEP) frequencies (supplemental Figure 3), as has been described on aggregate for *SF3B1*^K666N^.^10^

**Figure 1.**
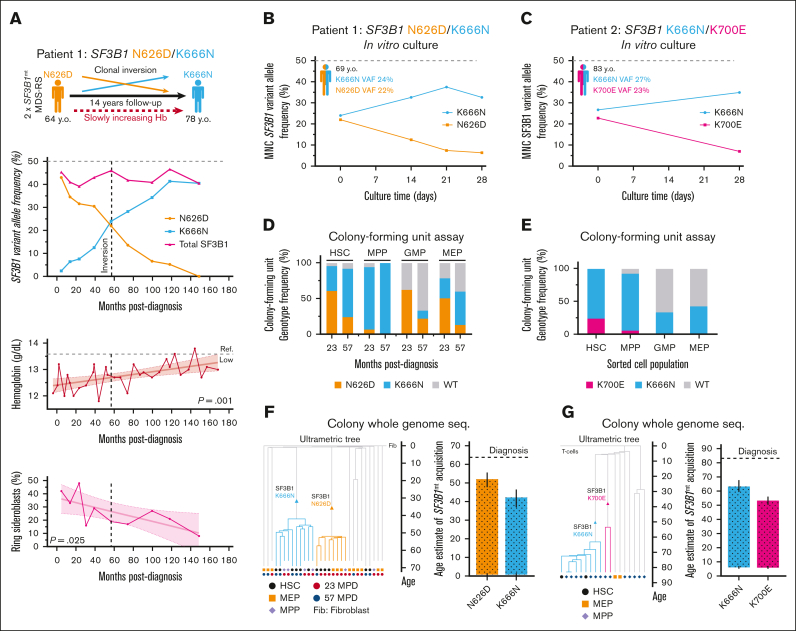
**Separate *SF3B1*-mutant clones arise independently decades before clinical disease and active clonal competition.** (A) Kinetics of ddPCR-assessed VAF of separate *SF3B1*^mt^ clones in BM mononuclear cells, clinical hemoglobin levels and BM ring sideroblast frequency of Patient 1, a patient with MDS-RS with separate *SF3B1* N626D/K666N clones followed up over 14 years. (B-C) ddPCR–assessed *SF3B1* VAFs from 3-dimensional *in vitro* culture time points of BM mononuclear cells seeded at (B) the inversion point of Patient 1’s long-term follow-up, and (C) the diagnostic visit of Patient 2, a patient with MDS-RS with separate *SF3B1* K700E/K666N clones. (D-E) Genotype frequencies of colonies derived from sorted HSPCs and cultured in colony-forming unit assays from Patient 1 (D) and Patient 2 (E). (F-G) Clonal composition dendrograms and estimated age intervals for acquisition of each *SF3B1* mutation by Patient 1 (F) and Patient 2 (G), relative to time of MDS-RS diagnosis. Error bars indicate 95% lower and upper bound errors for the age interval estimate. Comutations shared in all colonies of the same clone: P1-*SF3B1*^N626D^: *GPATCH1*^N787S^, *EEIG2*^R172S^, *RLBP1*^K270Q^; P1-*SF3B1*^K666N^: *INTS*^V124F^; P2-*SF3B1*^K666N^: *CPNE*^R517C^, *PCSK1*^E638K^, *SLC4A5*^P30S^, *FBXO34*^S320N^; P2-*SF3B1*^K700E^: *TTC12*^A389V^, *OR4N2*^T259M^, *KAT2A*^P691L^. ddPCR, digital droplet polymerase chain reaction; MPD, months post diagnosis.

To assess clone fitness in a context with minimal stromal, microenvironmental, and paracrine factors, we used an erythroid scaffold system^8^^,^^9^ for 28-day culture of BM mononuclear cells from Patient 1 at clonal inversion and from Patient 2 (VAF: *SF3B1*^K700E^, 23%; and *SF3B1*^K666N^, 27%). Despite a relatively short culture time, genotyping of both cultures demonstrated increased competition of *SF3B1*^K666N^ clones to the detriment of the coexisting *SF3B1*^mt^ clone (Figure 1B-C). Although clone-extrinsic elements may partially drive *SF3B1*^mt^ clonal expansion, these in vitro clonal competition dynamics support the hypothesis that the fitness advantage of *SF3B1*^K666N^ is mediated, at least in part, through a clone-intrinsic mechanism of hematopoietic origin.

Hematopoietic stem and progenitor cells (HSPCs) from both patients were then purified by fluorescence-activated cell sorting (supplemental Key Resources Table) to generate single-cell–derived HSPC colonies.^8^ DNA extraction and digital droplet polymerase chain reaction to assess colony genotypes showed that, whereas HSC-derived colonies closely mirrored total BM VAFs, *SF3B1*^K666N^ unexpectedly dominated multipotent progenitor–derived colony-forming units (Patient 1: month 23: 67 K666N/71 total [*P* < .0001]; Patient 1: month 57: 8/8 [not significant]; Patient 2: 105/121 [*P* < .0001]; Figure 1D-E; supplemental Table 2). Wild-type (WT) HSC/multipotent progenitor colony-forming units were near-absent and instead overrepresented in committed progenitors, likely because of *SF3B1*^mt^ differentiation defects.

Colony whole-genome sequencing (supplemental Methods) allowed us to reconstruct clonal hierarchies for each patient (Figure 1F-G). Although no candidate driver mutations were identified, most co-occurring mutations were present at an early age in both patients, and *SF3B1*^mt^ mutational rates were generally higher than reference WT data (supplemental Figure 4). Importantly, comparing the age intervals for *SF3B1*^mt^ acquisition (Patient 1, 0.5-50 years; Patient 2, 6-63 years) to age at diagnosis indicates that *SF3B1*^mt^ acquisition predated MDS onset by a minimum of 14 and 20 years, respectively. These MDS findings thus provide a direct parallel to the long clonal histories in myeloproliferative neoplasm development.^3^^,^^4^

Finally, the effect of *SF3B1*^mt^ on Patient 1’s HSPC compartment was evaluated using two single-cell RNA-sequencing techniques (Figure 2A; supplemental Methods): a pan-CD34 10x Genomics 3′ analysis capturing all HSPC compartments (Figure 2B; supplemental Figure 5) and genotype–targeted full-length TARGET sequencing (TARGET-seq)^11^ directed to HSC/MEP (Figure 2C; supplemental Figure 6). The median TARGET-seq splice junction coverage was ∼6500 splice junctions per cell (supplemental Figure 7A), enabling genotype-informed RNA splicing analyses. Despite obvious clonal outgrowth of *SF3B1*^K666N^, differential gene expression analysis of both data sets to identify shared patterns over time (10X) or mutation (TARGET-seq) identified only 6 matching differentially expressed genes, of which 4 were transcriptional regulators (Figure 2D).

**Figure 2.**
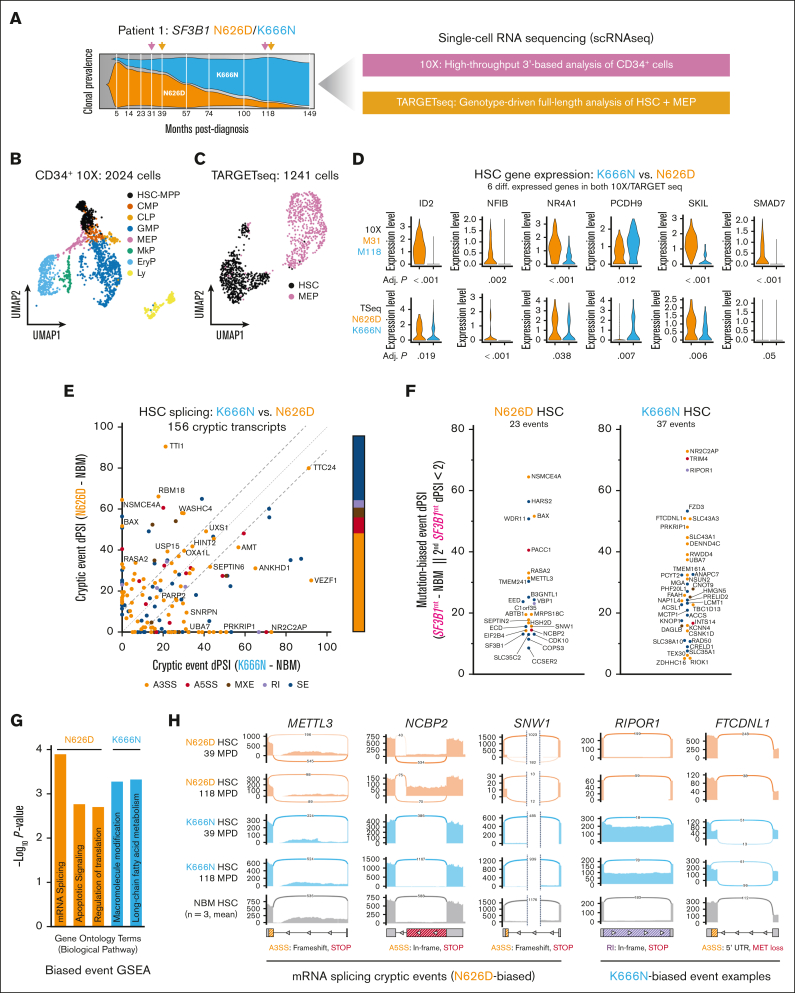
***SF3B1*^*mt*^ clone-intrinsic dominance is associated with differential transcript splicing but not differential gene expression.** (A) Distribution of scRNA-seq sampling times and experimental methods. (B-C) Uniform manifold approximation and projection (UMAP) of 10x Genomics-sequenced HSPCs (B) and TARGET-seq sequenced HSCs/MEPs (C). 10x HSPC subsets were annotated by comparing marker gene expression with existing literature data. The TARGET-seq annotation shown reflects the ground truth cell identification obtained from fluorescence-activated cell sorting purification. (D) Violin plots of common differentially expressed genes comparing month 118 and month 31 visits in the 10x HSC/MPP subset and between *SF3B1* K666N and N626D-genotyped HSC in TARGET-seq. Bonferroni-adjusted *P* values for the respective comparison are shown below each violin plot. (E) dPSI values comparing *SF3B1*^K666N^ and *SF3B1*^N626D^ HSC PSIs against control PSIs (NBM, n = 3). Event types are annotated per color. The diagonal lines highlight events with absolute clone/clone dPSI of <10. (F) PSI values of mutation-biased cryptic splicing events in *SF3B1*^N626D^ (left) and *SF3B1*^K666N^ HSC (right). A cryptic event was considered mutation-biased if the other mutant clone’s dPSI was <2%. (G) GO enrichment analysis of mutation-biased cryptic spliced genes in *SF3B1*^K666N^ and *SF3B1*^N626D^ HSC. (H) Sashimi plots of cryptic splicing events in *METTL3*, *NCBP2*, and *SNW1*, 3 of 4 genes associated with RNA splicing in the GO enrichment analysis; and *RIPOR1* and *FTCDNL1*, examples of K666N-biased splicing events. The predicted molecular consequences of the cryptic sites are annotated below the corresponding sashimi plots. A3SS, alternative 3′ splice site; A5SS, alternative 5′ splice site; CLP, common lymphoid progenitors; dPSI, percent spliced-in difference; EryP: erythroid progenitors; GO, Gene Ontology; Ly, lymphoid; MkP, megakaryocyte progenitors; MPP, multipotent progenitor; MPD, months post diagnosis; MXE, mutually exclusive isoform usage; NBM, normal BM; RI, intron retention; scRNA-seq, single-cell RNA sequencing; SE, exon skipping.

These restricted differences in gene expression (similar to previous reports)^7^ and the key splicing factor role of *SF3B1* led us to focus on RNA splicing patterns. Each *SF3B1*^mt^ clone was first compared against normal BM to delineate true RNA mis-splicing, primarily comprising alternative 3′ splice site usage and exon skipping events in both HSCs/MEPs (Figure 2E; supplemental Figure 7B-D; supplemental Table 3). Next, *SF3B1*^mt^ clones were compared with identify RNA mis-splicing biases. Several known *SF3B1*^mt^ mis-splicing events were indeed detected as common to HSCs/MEPs from both clones (eg, *OXA1L* and *SEPTIN6*; supplemental Figure 7E).^12^^,^^13^ Functional investigation of common HSC events (N626D/K666N, percent spliced-in difference of >2) identified several chromatin remodeling genes, including 2 critical to lymphoid development (*INO80D*^14^ and *CHD2*^15^); as well as R-loop formation elements^16^ (*ERCC3* and *TCEA2*; new: *LEO1*).

Mutation-specific filtering (second clone, percent spliced-in difference of <2) identified 60 exclusive mis-splicing events (Figure 2F). This included significant *SF3B1*^N626D^–unique truncating events affecting RNA splicing–associated genes (Figure 2G-H; per Gene Ontology enrichment, *METTL3*, *NCBP2*, *SNW1*, and *SF3B1*).^17^, ^18^, ^19^ Despite lacking a clear functional pattern, K666N events affected critical transcriptional regulators (eg, Myc pathway–associated *MGA* [not shown in figure] and *PHF20L1*; supplemental Figure 7E)^20^^,^^21^ with similar coding truncation (Figure 2H). Although the longitudinal study of only 1 patient limits the general inferences that can be made, our results lead us to hypothesize that (1) *SF3B1*^mt^ may induce epigenetic dysregulation in HSC via differential gene expression and mis-splicing of transcriptional regulators; and (2) “benign” *SF3B1* mutations (N626D/K700E) are partially so because of increased mis-splicing of splicing effectors, as has been indicated for the K700E hot spot.^22^ In contrast, the lack of this effect in *SF3B1*^K666N^ would increase relative fitness and the long-term likelihood of leukemic transformation.

Beyond the mechanisms explored here, other *SF3B1*^mt^ effects may also differ between mutations, such as R-loop accumulation and DNA damage,^23^ or protein changes downstream of aberrant RNA expression/splicing. However, our targeted investigation of the HSC compartment demonstrates that RNA mis-splicing is prevalent in the cell of disease origin, in which outgrowth capacity is most relevant.^8^ In conclusion, this work provides insight into long-term clonal competition patterns and enables a model for the relative advantages of *SF3B1*^mt^/WT and *SF3B1*^K666N^/*SF3B1*^mt^,^10^ highlighting the need for further investigation of the HSC compartment.

**Conflict-of-interest disclosure:** The authors declare no competing financial interests.

## Supplementary Material

Legends for Tables, Methods, Key Resources Table, Figures, and References
