# Supplementary material for: Competition of dual SF3B1mt clones in MDS-RS is associated with distinct RNA mis-splicing in hematopoietic stem cells
Source: Blood Neoplasia. 2024 Apr 12;1(2):100011. doi: 10.1016/j.bneo.2024.100011 (PMC12082108; doi:10.1016/j.bneo.2024.100011)
Supplement: Legends for Tables, Methods, Key Resources Table, Figures, and References [file BNEO_NEO-2024-000213-mmc1.pdf]

# Main supplemental file - Index

## Competition of dual *SF3B1*$^{mt}$ clones in MDS-RS is associated with distinct RNA mis-splicing in hematopoietic stem cells

Pedro Luis Moura†, Yasuhito Nannya†, Affaf Aliouat†, Isabel Juliana F Hofman†, Teresa Mortera-Blanco, Tetsuichi Yoshizato, Ryunosuke Saiki, Masahiro M Nakagawa, Ann-Charlotte Björklund, Gunilla Walldin, Indira Barbosa, Monika Jansson, Francesca Grasso, Maria Creignou, Edda M Elvarsdottir, Petter S Woll, Sten Eirik W Jacobsen*, Seishi Ogawa*, Eva Hellström-Lindberg*

† *PLM, YN, AA and IJH are joint first authors and contributed equally to this study.*

** **Corresponding Author 1**: Eva Hellström-Lindberg, M.D., PhD; e-mail: eva.hellstrom-lindberg AT ki.se; Phone: +46-8-585 800 00*

*Address: Karolinska Institutet, Center for Hematology and Regenerative Medicine, Department of Medicine Huddinge, Karolinska University Hospital, SE-141 83 Huddinge, Sweden.*

** **Corresponding Author 2**: Seishi Ogawa, M.D., PhD; e-mail: sogawa-tky AT umin.ac.jp; Phone: +81-75-753-9285*

*Address: Kyoto University, Department of Pathology and Tumor Biology, Yoshida-honmachi, Sakyo-ku, Kyoto 160-8582, Japan.*

** **Corresponding Author 3**: Sten Eirik W. Jacobsen, M.D., PhD; e-mail: sten.eirik.jacobsen AT ki.se; Phone: +46-072 858 3119*

*Address: Karolinska Institutet, Center for Hematology and Regenerative Medicine, Department of Medicine Huddinge, Karolinska University Hospital, SE-141 83 Huddinge, Sweden.*

Supplemental Table Legends ........ 2
Supplemental Methods ........ 3
Key Resources Table ........ 5
Supplemental Figures ........ 8
  Supplemental Figure 1 ........ 8
  Supplemental Figure 2 ........ 9
  Supplemental Figure 3 ........ 10
  Supplemental Figure 4 ........ 11
  Supplemental Figure 5 ........ 12
  Supplemental Figure 6 ........ 14
  Supplemental Figure 7 ........ 16
References ........ 18

# Supplemental Table Legends

**Supplemental Table 1)** Summary data of dual *SF3B1*$^{mt}$ patient frequencies among the Karolinska Institutet and Kyoto University clinical cohorts, splice factor mutational overview, longitudinal clinical variables of Patient 1's follow-up and map of experiments performed in this work.

**Supplemental Table 2)** Colony-forming unit count, digital droplet PCR-identified genotype and DNA yield. Colonies in green were submitted for whole genome sequencing due to presenting a clear genotype of single-cell origin and sufficient total DNA yield.

**Supplemental Table 3)** Cryptic *SF3B1*$^{mt}$ splicing events identified through rMATS analysis of TARGET-seq transcriptomic data, separated by splicing event type (A3SS: alternative 3' splice site; A5SS: alternative 5' splice site; MXE: mutually exclusive isoform usage; RI: intron retention; SE: exon skipping), joined altogether (Total) and separated by cell type (HSC or MEP). P-values were calculated using the likelihood-ratio test (LRT), adjusted with the Benjamini-Hochberg method and used for quality control of the detected cryptic junctions (Adj. P-val < 0.001, min. canonical/cryptic junction reads > 50, dPSI cut-off > 0.20, PSI standard deviation < 0.25).

# Supplemental Methods

**Statistical methods**
Statistical methods specific to high-throughput data formats are detailed below. scRNAseq analysis was performed using Seurat v.3.1.4 with RStudio Server v. 1.3.1056 and R v. 3.6.3. All other statistical analyses were performed with RStudio v. 1.4.1767, R v. 4.0.5, Excel v. 2204 and GraphPad Prism v. 9.4.0.

**Whole-genome sequencing (WGS)**
A minimum of 50 ng of purified DNA obtained from single cell-derived colonies were subjected to library preparation using a KAPA Hyper Prep Kit. The libraries from Patient 1 and Patient 2 were sequenced using an Illumina NovaSeq 6000 (Illumina) and a DNBSEQ-G400 (MGI Tech), respectively. The target depth was set at 15x on paired-end mode with read length 150 bp. The Genomon2 pipeline was used for mutation calling (vs. the GRCh37 reference genome) with default settings and significant called mutations were selected (the EBCall P-value and Fisher's P-value were $\leq 10^{-6}$, $\leq 10^{-2}$) after removal of major SNPs (1000g2014oct_all >0.1%, ExAC >0.001).[1] SNV/indel calling was performed only on autosomes. Colonies that did not follow a binomial distribution with a median VAF = 0.5 and colonies with two *SF3B1* mutations were examined for removal due to suspected contamination. Because low-depth WGS has many false-negative calls, a sum set of variant calls from all colonies was created, and the number of variant and wildtype reads of this set was then generated for all colonies. Assuming a binomial distribution, a mutant was judged as mutant when the likelihood of being mutant was at least 20 times higher than the likelihood of being wildtype, and a wildtype was judged to be wildtype when the likelihood of being wildtype was at least 20 times higher than the likelihood of being mutant. Other mutations were considered undecidable, thus constructing a genotype matrix. We excluded variants determined to be undecidable in more than 30% of colonies. The phylogenetic trees were topologically determined using maximum parsimony tree inference and bootstrap approximation with MPBoot.[2] We assigned mutations back to branches of the phylogenetic tree using treemut (https://github.com/NickWilliamsSanger/treemut). Variant call sensitivity was estimated from germline variants and the tree lengths were adjusted by scaling 1/sensitivity. Ultrametric trees were constructed, including estimates for the time of *SF3B1* mutation acquisition, using the Phylodyn package.[3]

**10X Single-cell RNA sequencing and analysis**
All samples were loaded onto Chromium Single Cell Chips (10x Genomics, CA, USA) at a target capture rate of 10,000 cells per sample. Single cell libraries were prepared using Chromium Next GEM Single Cell 3' Kits v3.1 (10x Genomics) as per the manufacturer's instructions. Libraries were pooled and sequenced on an Illumina NextSeq 2000 (Illumina), read length 100 bp. Read pseudoalignment was performed against the GRCh38.p13 human genome assembly[4] through kallisto v0.46.1 and bustools v0.40.0[5] was used for barcode and unique molecular identifier (UMI) counting. Seurat v3.1.4[6] was used to load, process, and analyze the resulting count matrices. For differential gene expression analyses, quality control steps were first performed through removal of cells expressing less than 200 distinct genes, total percentage of mitochondrial reads above 15% and a total read count below 2000 counts, as well as through removal of genes expressed in less than 3 cells (**Sup Fig 4**). All samples were subjected to Seurat's NormalizeData function through the LogNormalize method, and the 2000 most highly variable genes across all samples were identified and used as a basis for anchor identification and integrated with literature BM datasets for cell type mapping.[7,8] After integration, the data were scaled and subjected to Principal Component Analysis (PCA). The top 50 PCs with lowest P-values were selected for UMAP projection[9] and clustering (through a shared nearest neighbor [SNN] modularity optimization-based clustering algorithm[10] at a resolution parameter of 0.6). The dataset was filtered to include only CD34-expressing cells, and clustering was performed again at a resolution parameter of 1.8. The generated clusters were then manually annotated by comparison of their gene expression profiles with literature-defined expression patterns and through analysis of the cluster marker genes in Enrichr.[11] Differentially expressed genes between Patient 1's CD34-expressing cells from Month 39 and Month 118 were identified through Seurat's non-parametric Wilcoxon rank sum test (adjusted P-value < 0.10).

**Single-cell TARGET-seq cDNA synthesis and genotyping**
cDNA synthesis and library preparation were performed as described in Rodriguez-Meira A. *et al.*,[12] using 24 cycles of PCR amplification. Target-specific primers spanning patient-specific mutations were added to reverse transcription (RT) and PCR steps. Specifically, these primers targeted DNA (gDNA) and transcript (mRNA) amplicons for a wildtype *SF3B1* sequence (SF3B1), *SF3B1* K666N (K666N_Geno) and *SF3B1* N626D (N626D_geno). Additional primers targeted clone-specific co-mutations through amplification of wildtype and mutant sequences of the genes *RLBP1* (RLBP1, RLBP1_geno), *CAMTA1* (CAMTA1, CAMTA1_Geno) and GNK1 (GNK1, GNK1_Geno). All primer sequences are listed in the key resources table above. After cDNA synthesis, half of the cDNA from each single-cell library was purified and sequenced using a NextSeq 500 instrument (Illumina) with custom sequencing primers and following the following sequencing configuration: 150 bp Read1; 10 bp Index read; 150 bp Read 2. The FASTQ file containing targeted gDNA and cDNA-derived sequencing reads from each

single cell was aligned to the human reference genome (GRCh37/hg19) using BWA v0.7.17[13] and STAR v2.6.1d[14]. Samtools v1.9[15] was used to concatenate the BAM header, and subsequently both gDNA and mRNA reads were tagged with cell identifiers using Picard v2.3.0 "*AddOrReplaceReadGroups",* and duplicate reads marked using "*MarkDuplicates"*. mRNA sequencing reads overhanging into intronic regions were hard-clipped using GATK[16] v4.1.2.0 *SplitNCigarReads*. Variant calling was performed using GATK *Mutect2*. The frequency of each nucleotide (A, C, G, T) and indels at each pre-defined variant site were also called using Samtools *mpileup*. Lastly, the coverage at each pre-defined variant site was calculated using Bedtools v2.27.1.[17] A cut-off coverage outlier value was computed for each variant as having a coverage exceeding 1.5 times the length of the interquartile range from the 75th percentile the coverage of empty wells (blank control). Next, a value of 30 was added to this outlier value to yield the final coverage threshold to be used for genotype assignment. Variant site assigned genotypes were based on the highest score. A Chi-square ($\chi^2$) test was first used to compare the observed frequency of reference and alternative alleles against the expected fraction of reference and alternative alleles. $\chi^2$ statistics were then tabulated for each fitted model and converted to genotype scores. Variant sites with 2% < VAF < 4% and 96% < VAF < 98% were assigned as "ambiguous". Genotyping was based the above-described gDNA amplicons from *SF3B1* N626D and K666N mutations, as well as gDNA amplicons from clone-specific co-mutations (*RLBP1*$^{K261Q}$ [N626D], and *CAMTA1*$^{intronic}$ / *GNK1*$^{intronic}$ [K666N]) to enhance the reliability of wildtype calls. Wildtype genotypes were assigned to cells with no mutant variants detected and sufficient read coverage, with cells below the read coverage limit being assigned as "low coverage". Variants with ambiguous or low coverage assignments for a particular cell were excluded from analysis. Importantly, the low number of cells with conflicting genotypes (<1%) and biallelic mutant, which is rare in MDS, and the absence of *SF3B1* mutations in cells originating from the normal healthy control donors validated the high reliability in the genotype assignment of the cells originating from the MDS-RS patient.

**TARGET-seq transcriptome library preparation and sequencing**

Half of the TARGET-seq cDNAs were used for transcriptomic analysis. After cDNA tagmentation and indexing, 384-well plate single-cell library cDNAs were pooled, purified and resuspended in a final volume of 50 µL of EB buffer (Qiagen). Each indexed library was diluted to 4 nM. Libraries were sequenced on a NovaSeq 6000 instrument (Illumina), with the following sequencing configuration: 150 bp Read 1, 8bp Index 1 (i7), 8 bp Index 2 (i5), 150 bp Read 2. Nucleotide bases from the 3'-end of the reads with Phred score <20 were trimmed using TrimGalore v. 0.6.5 [18]. Trimmed reads were mapped to the human reference genome (GRCh38.p13) using STAR v. 2.6.1d in 2-pass mode[14]. Splice junction counts were collated into a matrix of splice junction coordinates per cell ID. Gene expression was quantified in raw counts using featureCounts v. 2.0.1[19] or in transcripts per million (TPM) using RSEM v.1.2.31.[20] Seurat v3.1.4[6] was used to load, process, and analyze the raw count matrices. Reads were normalized by sequencing plate through Seurat's SCTransform function, and quality control followed through removal of cells expressing less than 200 distinct genes, total percentage of mitochondrial reads above 10% and a total transcript count below 50000 counts, as well as through removal of genes expressed in less than 3 cells (**Sup Fig 5**). After integration, the data were scaled, PCs were calculated based on 3000 variable features and the 50 PCs with lowest P-values were selected for UMAP projection[9] and clustering (through a SNN modularity optimization-based clustering algorithm[10] at a resolution parameter of 1.8). Differentially expressed genes between Patient 1 HSC from K666N and N626D were identified through Seurat's non-parametric Wilcoxon rank sum test (adjusted P-value < 0.10).

**TARGET-seq splicing event detection and quantification**

Cell-specific BAM files were merged per cell type, visit and donor of origin using Samtools *merge* to generate pseudobulk BAM files. Differential splicing analysis was performed between cell type-mutation groups (N626D-HSC, N626D-MEP, K666N-HSC, K666N-MEP, NBM-HSC, NBM-MEP, with visits or biological donors used as replicates) using rMATS v. 4.1.1.[21] P-values were calculated using the likelihood-ratio test (LRT), adjusted with the Benjamini-Hochberg method and used for quality control of the detected cryptic junctions (Adj. P-value < 0.001, min. canonical/cryptic junction reads > 50, dPSI cut-off > 0.20, PSI standard deviation < 0.25). Cryptic splice site distances from the canonical region were calculated via determining the absolute difference between cryptic and canonical sequences (long/short in rMATS output, and exon start/end depending on chromosomal strand). Sashimi plots for visualization were generated using ggsashimi v. 1.1.5.[22] Single-cell PSIs were quantified and compared between mutation groups using MARVEL[23]. Gene ontology enrichment analysis of mutation-specific mis-spliced genes was performed using the Enrichr[11] GO Biological Pathways 2023 module, at an adj. P-value cut-off of 0.10.

# Key Resources Table

| REAGENT or RESOURCE | SOURCE | IDENTIFIER |
|---|---|---|
| **Antibodies** | | |
| CD45RA-BV421 | Invitrogen | Cat no. 404-0458-42, RRID:AB_2925509 |
| CD90-PE | Biolegend | Cat no. 328110, RRID:AB_893442 |
| CD3-PE-Cy5 | Biolegend | Cat no. 300410, RRID:AB_314064 |
| CD4-PE-Cy5 | Biolegend | Cat no. 300510, RRID:AB_314078 |
| CD7-PE-Cy5 | Biolegend | Cat no. 343110, RRID:AB_2075096 |
| CD8a-PE-Cy5 | Biolegend | Cat no. 301010, RRID:AB_314127 |
| CD10-PE-Cy5 | Biolegend | Cat no. 312206, RRID:AB_314917 |
| CD11b-PE-Cy5 | Biolegend | Cat no. 301308, RRID:AB_314160 |
| CD14-PE-Cy5 | Beckman Coulter | Cat no. A07765, RRID:AB_3068601 |
| CD19-PE-Cy5 | Biolegend | Cat no. 302210, RRID:AB_314239 |
| CD20-PE-Cy5 | Biolegend | Cat no. 302308, RRID:AB_314255 |
| CD56-PE-Cy5 | BD Biosciences | Cat no. 555517, RRID:AB_395907 |
| CD235ab-PE-Cy5 | Biolegend | Cat no. 306605, RRID:AB_314623 |
| CD123-PE-Cy7 | Biolegend | Cat no. 306010, RRID:AB_493576 |
| CD38-PE-Dazzle594 | Biolegend | Cat no. 303538, RRID:AB_2564104 |
| CD38-PE-TexasRed | Invitrogen | Cat no. MHCD3817, RRID:AB_10392545 |
| CD34-BUV395 | BD Biosciences | Cat no. 745727, RRID:AB_2743203 |
| CD45RA-BB515 | BD Biosciences | Cat no. 564552, RRID:AB_2738841 |
| CD41a-APC | BD Biosciences | Cat no. 561852, RRID:AB_10895580 |
| **Nucleotide sequences** | | |
| gDNA_SF3B1_Fw | This paper | ATCTCCCCAAATCAGTAGCCCAA |
| gDNA_SF3B1_Rev | This paper | ATTACCAACTCATGACTGTCCTTTC |
| mRNA_SF3B1_Fw | This paper | ACCATAAGGAGTTGCTGCTTCA |
| mRNA_SF3B1_Rev | This paper | CGTGGTCATTGAACCGCTATTG |
| gDNA_K666N_Geno_Fw | This paper | TTAAACATGGACAGGCTGTGTG |
| gDNA_K666N_Geno_Rev | This paper | CTTTTGCTGTTGTAGCCTCTGC |
| mRNA_K666N_Geno_Fw | This paper | AGCACTGATGGTCCGAACTT |
| mRNA_K666N_Geno_Rev | This paper | GCCCTGGGCATTCCTTCTTTA |
| gDNA_N626D_Geno_Fw | This paper | TACCAGTGTGTCTCGCTTGC |
| gDNA_N626D_Geno_Rev | This paper | TGTTTACATTTTAGGCTGCTGG |
| mRNA_N626D_Geno_Fw | This paper | CTCGCTTGCCAGGACTTCTT |
| mRNA_N626D_Geno_Rev | This paper | CTAGAGTGGAAGGCCGAGAGAT |
| gDNA_RLBP1_Fw | This paper | GGGGCTGATGGACAATGAGG |
| gDNA_RLBP1_Rev | This paper | CATGTTGGGTGTCAGTGGGAT |
| mRNA_RLBP1_Fw | This paper | ACAAGTATGGCCGAGTGGTC |
| mRNA_RLBP1_Rev | This paper | TGAGGAGAGGCCCAGAGATT |
| gDNA_RLBP1_Geno_Fw | This paper | GGGTATCTCTCTGTCACCGC |
| gDNA_RLBP1_Geno_Rev | This paper | GGACCATGGTAGAGTGTGAGG |
| mRNA_RLBP1_Geno_Fw | This paper | GGTGGACATGCTCCAGGATT |
| mRNA_RLBP1_Geno_Rev | This paper | CATCATACTTGGGCAGCGTG |
| gDNA_CAMTA1_Fw | This paper | GAAGGGAGCTGAGCCAGTTG |
| gDNA_CAMTA1_Rev | This paper | GTTAACCGCTGCTAATGGGG |
| gDNA_CAMTA1_Geno_Fw | This paper | GGAGCACAAGCTGTAGCCAG |
| gDNA_CAMTA1_Geno_Rev | This paper | CATTCGTGTTCTCGTTCTGCC |
| gDNA_GNK1_Fw | This paper | ACACTGCTAGTGACATCGCC |
| gDNA_GNK1_Rev | This paper | CAGGTTGTTGATGCTCACCT |
| gDNA_GNK1_Geno_Fw | This paper | TGGCATCCAAACTGCCTACT |
| gDNA_GNK1_Geno_Rev | This paper | TGAATTTTGGTCAGTGCCCC |
| ddPCR FAM/HEX primers, *SF3B1* p.N626D c.1876A>G | Bio-Rad | ID no. dHsaMDS539025266 |
| ddPCR FAM/HEX primers, *SF3B1* p.K666N c.1998G>C | Bio-Rad | ID no. dHsaMDS316001838 |

| | | |
|---|---|---|
| ddPCR FAM/HEX primers, *SF3B1* p.K700E c.2098A>G | Bio-Rad | ID no. dHsaMDS576883070 |
| A1+A2:A3+A4 barcoded oligodT | Biomers | Custom primers |
| **Chemicals** | | |
| 7-amino-actinomycin D | eBiosciences | Cat no. 00-6993-50 |
| Lymphoprep™ | STEMCELL Technologies | Cat no. 07851 |
| RPMI 1640 Medium, GlutaMAX™ | Thermo Fisher | Cat no. 61870143 |
| Heat-inactivated fetal bovine serum | Thermo Fisher | Cat no. 10082147 |
| Dimethyl Sulfoxide | Sigma-Aldrich | Cat no. D2650 |
| DNase I | Sigma-Aldrich | Cat no. D4513 |
| Phosphate Buffer Saline (PBS) | Thermo Fisher | Cat no. 10010031 |
| Ethylenediaminetetraacetic acid (EDTA) | Sigma-Aldrich | Cat no. 03690 |
| MethoCult H4434 | STEMCELL Technologies | Cat no. 4434 |
| StemSpan™ SFEM | STEMCELL Technologies | Cat no. 09600 |
| Triton 0.43% | Sigma-Aldrich | Cat no. T8787 |
| RNAse Inhibitor | TAKARA Clontech | Cat no. 2313A |
| dNTPs (10 mM) | Life Technologies | Cat no. R0192 |
| Protease (1.09 AU/mL in H2O) | Qiagen | Cat no.19155 |
| ERCC (E5) | Life Technologies | Cat no. 4456740 |
| EB buffer | Qiagen | Cat no. 19086 |
| Iscove's Modified Dulbecco's Medium (IMDM) | Sigma-Aldrich | Cat no. I3390-500ML |
| GlutaMAX™ | Thermo Fisher | Cat no. 35050061 |
| Penicillin/Streptomycin | Thermo Fisher | Cat no. 15140122 |
| BIT9500 serum substitute | StemCell Technologies | Cat no. 9500 |
| Recombinant human stem cell factor | Invitrogen | Cat no. 300-07 |
| Recombinant human interleukin 3 | Invitrogen | Cat no. 200-03 |
| Recombinant human interleukin 6 | Invitrogen | Cat no. 200-06 |
| Recombinant human erythropoietin | Nordic Biosite | Cat no. PAT-FKJJ4L-500 |
| Iron saturated human holo-Transferrin | Sigma-Aldrich | Cat no. T0665-50MG |
| Calf skin collagen type I | Sigma-Aldrich | Cat no. C9791 |
| 1,4-Dioxane 99.8% | Sigma–Aldrich | Cat no. 5895911000 |
| Polyurethane | Noveon | Cat no. EG-80A |
| **Critical Commercial Assays** | | |
| CD34 MicroBeads | Miltenyi Biotec | Cat no. 130-046-702 |
| ddPCR™ Supermix for Probes (No dUTP) | Bio-Rad | Cat no. 1863023 |
| KAPA Hyper Prep Kit | Roche | Cat no. 07962312001 |
| Ampure XP Beads | Beckman Coulter | Cat no. A63881 |
| Nextera XT DNA Library Preparation Kit | Illumina | Cat no. FC-131-1096 |
| Nextera XT Index Kit v2 Set A (Index Kit v2 Set A | Illumina | Cat no. FC-131-2001 |
| Nextera XT Index Kit v2 Set D | Illumina | Cat no. FC-131-2004 |
| **Software and Algorithms** | | |
| FlowJo v. 10.7.2 | BD Biosciences | RRID:SCR_008520 |
| RStudio v. 1.4.1767 | Posit | RRID:SCR_000432 |
| R v. 4.0.5 | R Project for Statistical Computing | RRID:SCR_001905 |
| Microsoft Excel v. 2204 | Microsoft | RRID:SCR_016137 |
| GraphPad Prism v. 9.4.0 | GraphPad | RRID:SCR_002798 |
| Seurat v. 3.1.4 | [6] | RRID:SCR_007322 |
| BWA v. 0.7.17 | [13] | RRID:SCR_010910 |
| STAR v. 2.6.1d | [14] | RRID:SCR_004463 |
| Picard v. 2.3.0 | Broad Institute | RRID:SCR_006525 |

| | | |
|---|---|---|
| GATK v. 4.1.2.0 | [16] | RRID:SCR_001876 |
| SAMtools v. 1.9 | [15] | RRID:SCR_002105 |
| TrimGalore v. 0.6.5 | [18] | N/A |
| EnrichR v. | [11] | N/A |
| QuantaSoft v. 1.7.4 | Bio-Rad | N/A |
| Genomon2 v. 2.6 | Human Genome Center | N/A |
| **Deposited Data** | | |
| Colony whole-genome sequencing of Patient 1 and 2 | This paper | DOI: 10.48723/tt0e-eq82 |
| 10X single-cell RNAseq of Patient 1 (M31 and M118) and 1 NBM donor | This paper | DOI:10.48723/tt0e-eq82 |
| TARGET-seq combined genotype/RNAseq of Patient 1 (M39 and M118) and 3 NBM donors | This paper | DOI:10.48723/d7s9-6336 |

# Supplemental Figures

## Supplemental Figure 1

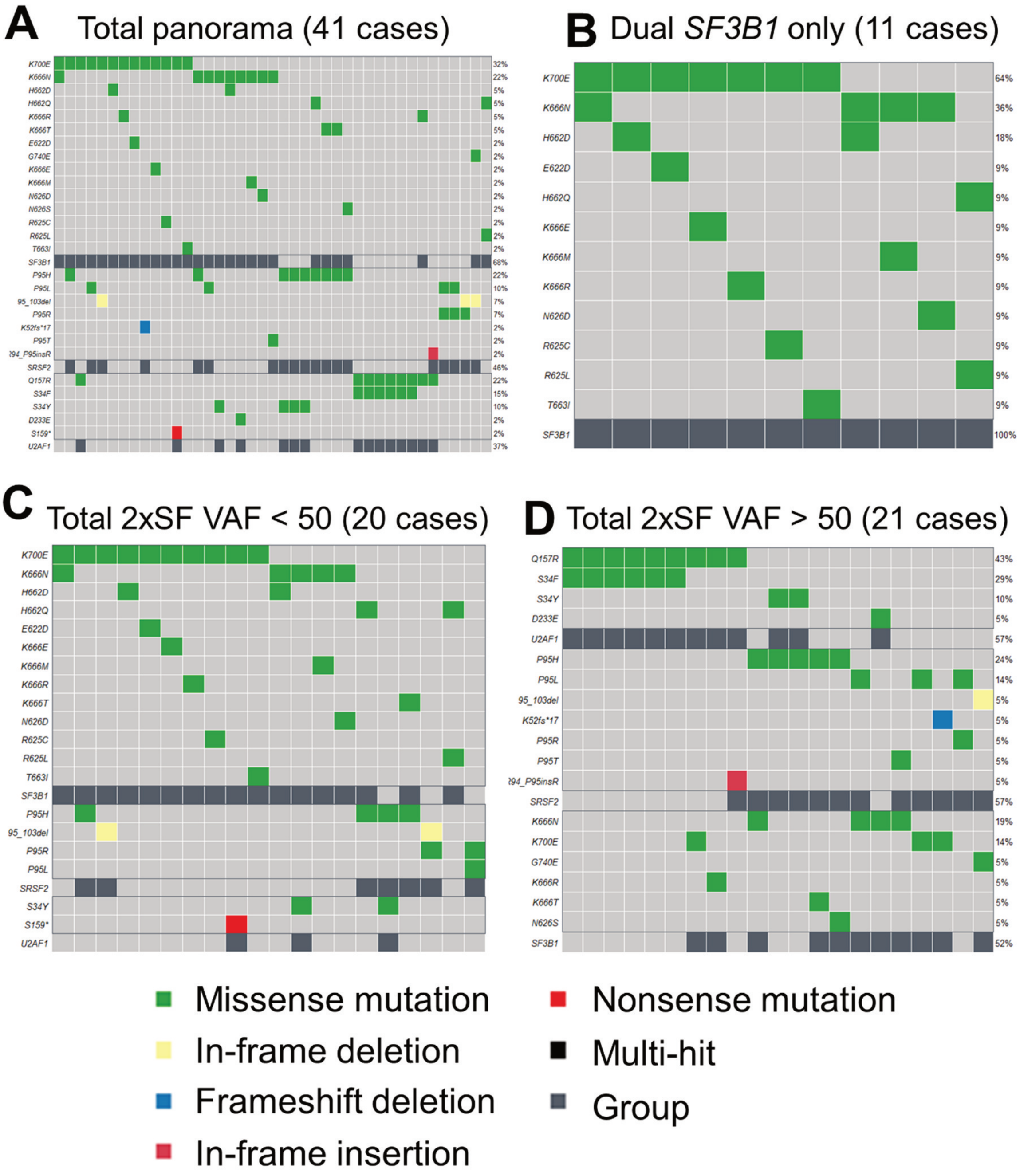


**Sup. Fig. 1) Genetic panorama of 2xSFmut patients in the Karolinska Institutet and Kyoto University clinical cohorts**

Total panorama (**A**), dual *SF3B1*mt cases (**B**), likely dual SFmt cases (**C**) and potential co-occurring SFmt cases (**D**), separated by mutant site and splice factor (*SF3B1*, *SRSF2*, *U2AF1*). Oncoplots were generated using maftools.[24]

## Supplemental Figure 2

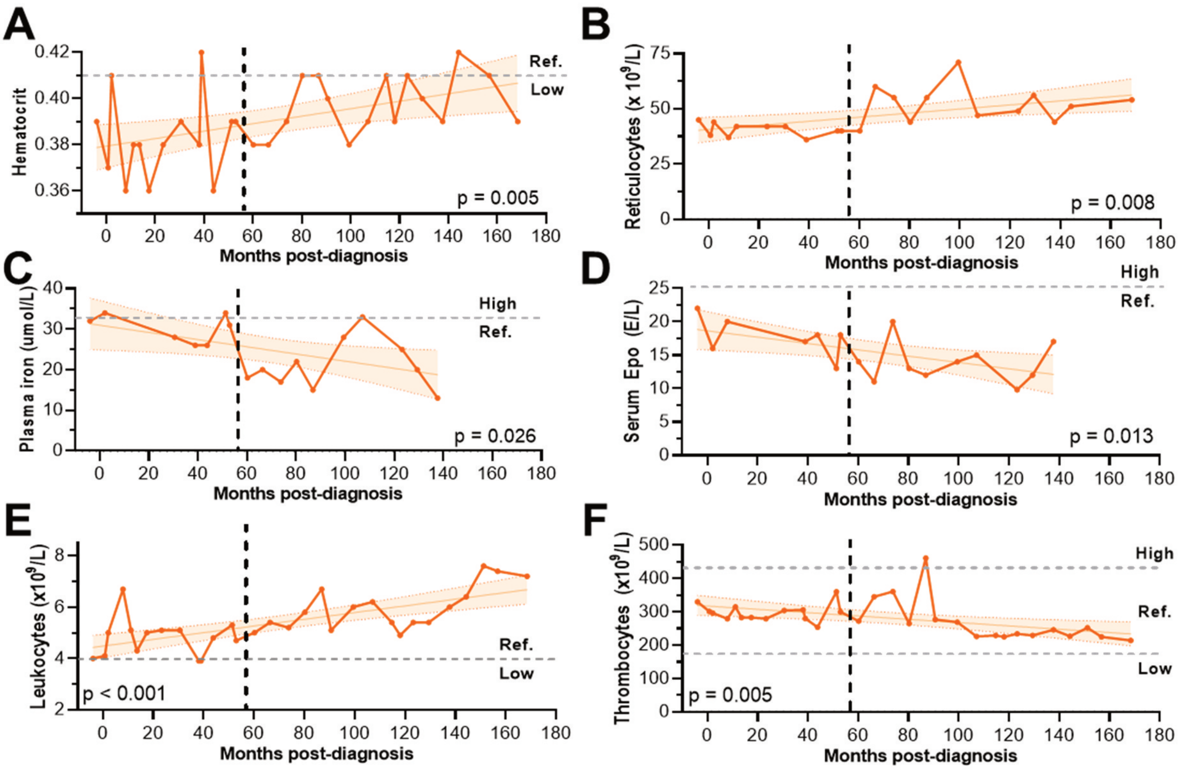


**Sup. Fig. 2) Changes in peripheral blood hematological parameters during Patient 1's clonal inversion**

Kinetics of hematocrit values (**A**), reticulocyte counts (**B**), plasma iron (**C**), serum erythropoietin (Epo) (**D**), leukocyte counts (**E**) and thrombocyte counts (**F**). P-values were calculated via F test to assess significant slope deviation from zero.

## Supplemental Figure 3

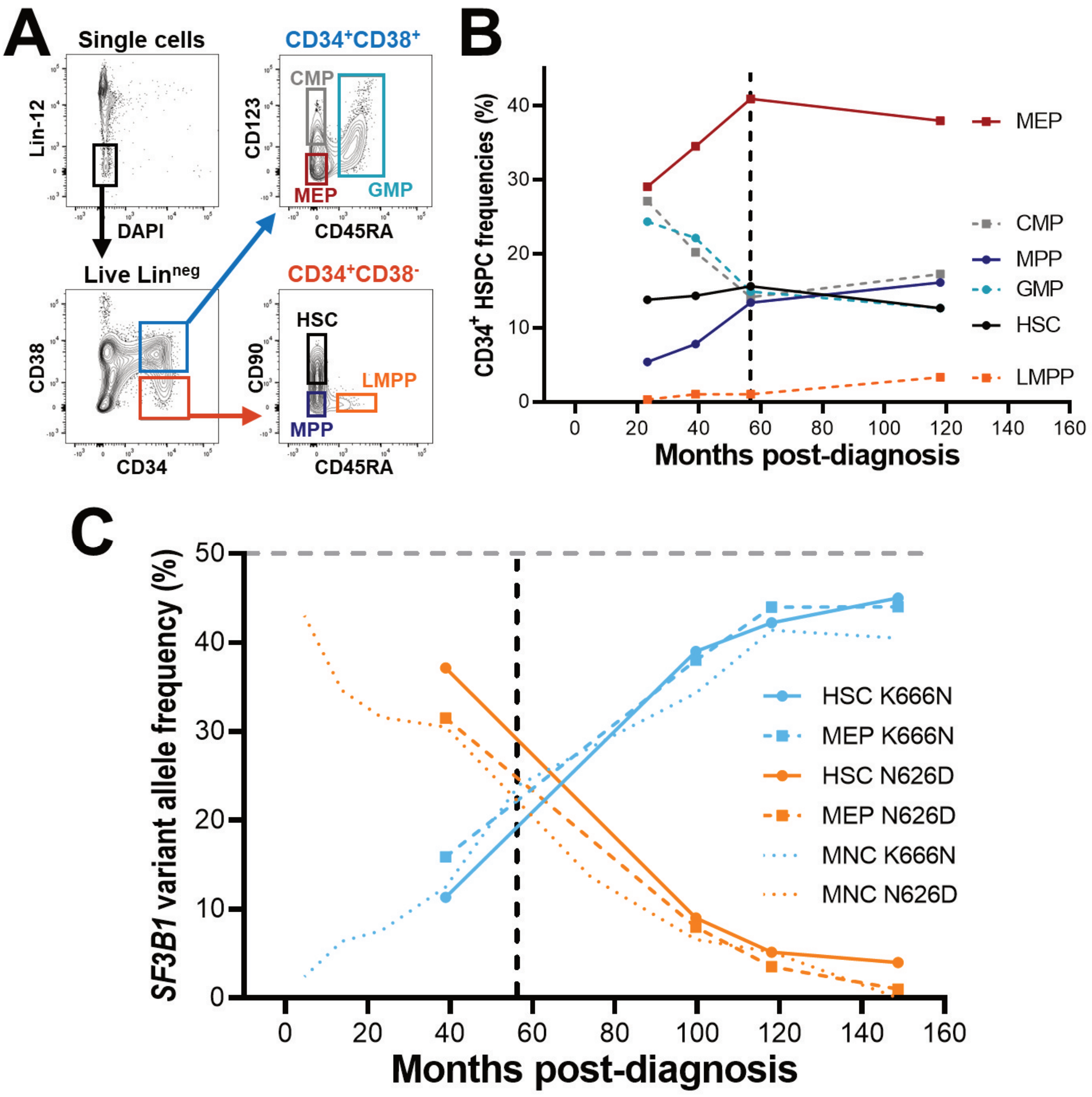


**Sup. Fig. 3) Hematopoietic stem and progenitor cell composition and dynamics of Patient 1**

**A)** FACS strategy for CD34+ hematopoietic stem and progenitor cell (HSPC) subset isolation.

HSC: Hematopoietic stem cells; MPP: Multipotent progenitors; LMPP: Lymphoid-primer multipotent progenitors; CMP: Common myeloid progenitors; MEP: Megakaryocyte-erythroid progenitors; GMP: Granulocyte-monocyte progenitors.

**B-C**) Patient 1 time-course of (B) relative HSPC subset frequencies of total CD34+CD38+/- cells and (C) ddPCR-assessed *SF3B1* VAFs in sorted HSCs, MEPs and total MNCs.

## Supplemental Figure 4

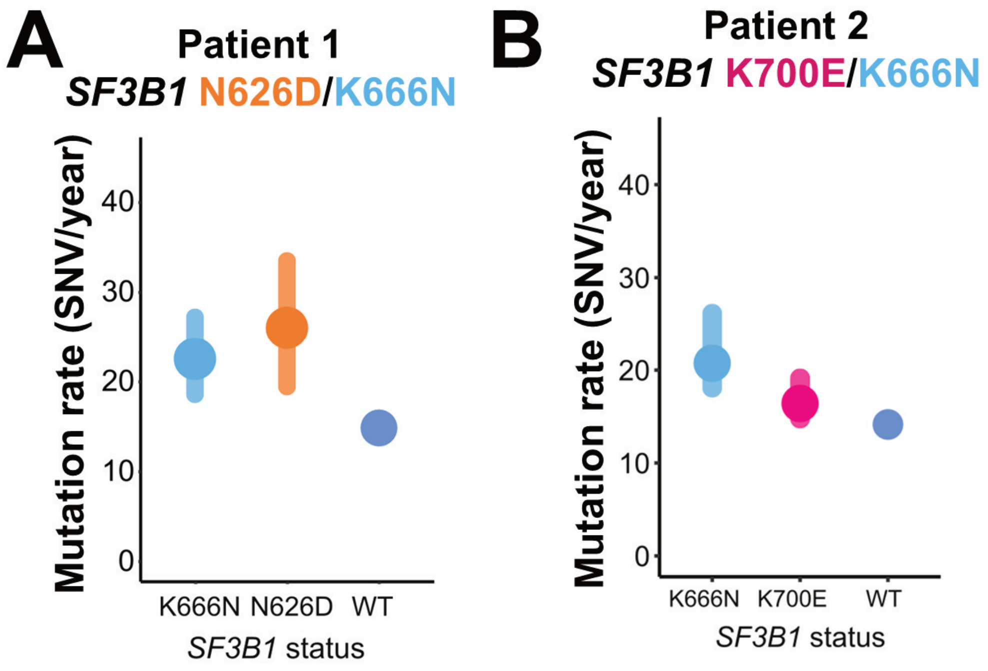


**Sup. Fig. 4) Enhanced mutational rate of *SF3B1*$^{mt}$ clones**

**A**/**B)** Rate of single nucleotide variant (SNV) acquisition (mutation rate) in each *SF3B1*$^{mt}$ clone in Patient 1 (B) and Patient 2 (C), compared to reference database normal control (*SF3B1*-wildtype) data (WT). Vertical lines indicate a 95% confidence interval for the calculated mutation rates.

## Supplemental Figure 5

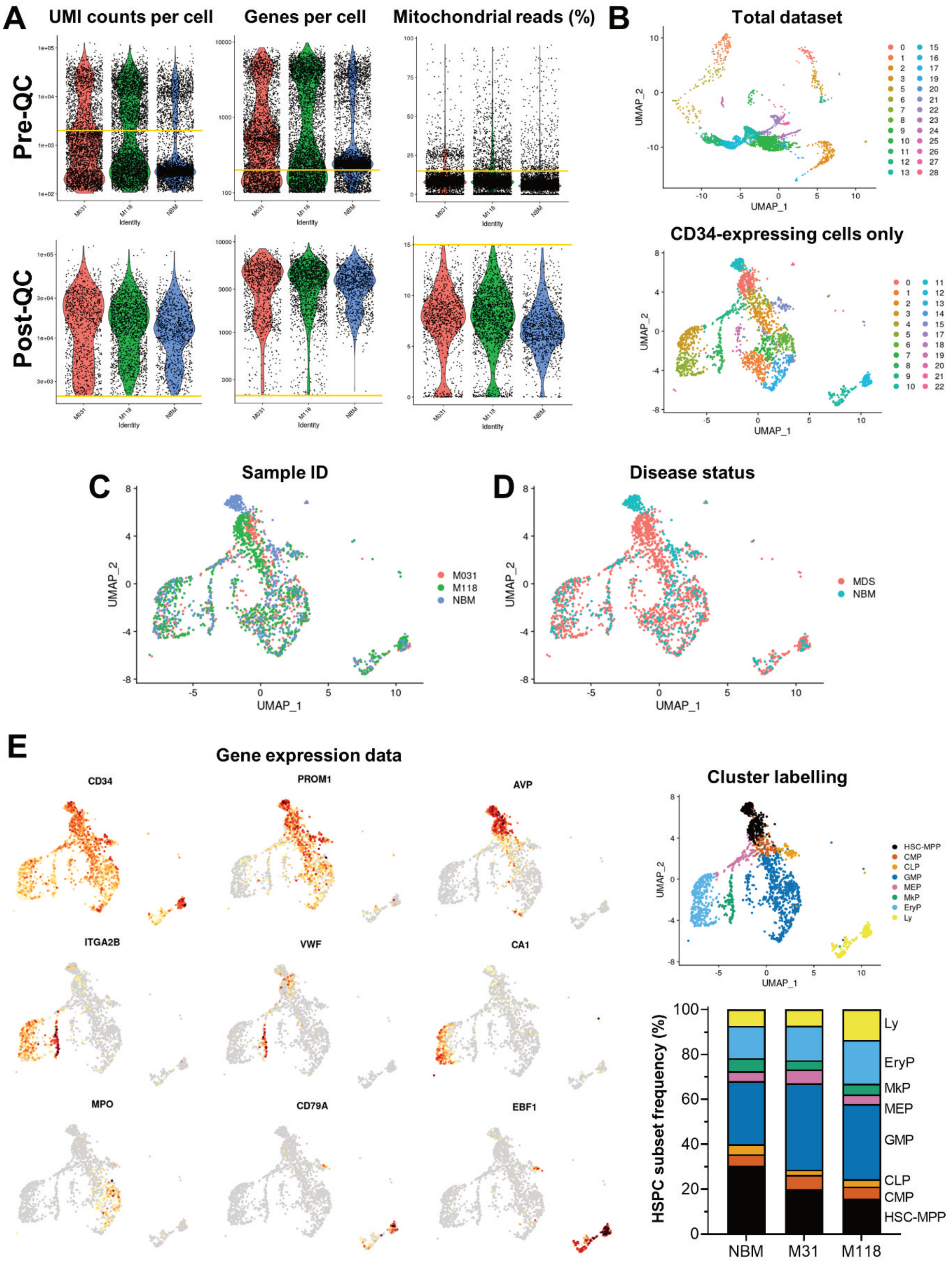


**Sup. Fig 5) Quality control and baseline metrics of 10X transcriptional data**

**Sup. Fig 5) Quality control and baseline metrics of 10X transcriptional data**

**A)** Violin plots displaying Unique Molecular Identifier (UMI) counts per cell, obtained gene number per cell and mitochondrial read proportion per cell in 10X single-cell RNAseq data, before (top row) and after (bottom row) quality control cut-offs (Min. 2000 UMI per cell; Min. 200 genes per cell; Max 15% mitochondrial reads per cell).

**B)** UMAP of the total collected dataset (2 visits at Month 31 and Month 118 from Patient 1 and 1 NBM control), before (top) and after (bottom) cut-off to exclude cells without detected CD34 transcripts.

**C/D)** UMAP of CD34-expressing cells, separated by sample ID (**C**) and disease status (**D**).

**E)** UMAP overlays with selected marker gene expression, cluster labelling identification based on the selected marker genes (top right) and comparison of cell type frequencies per 10X sample (bottom right).

## Supplemental Figure 6

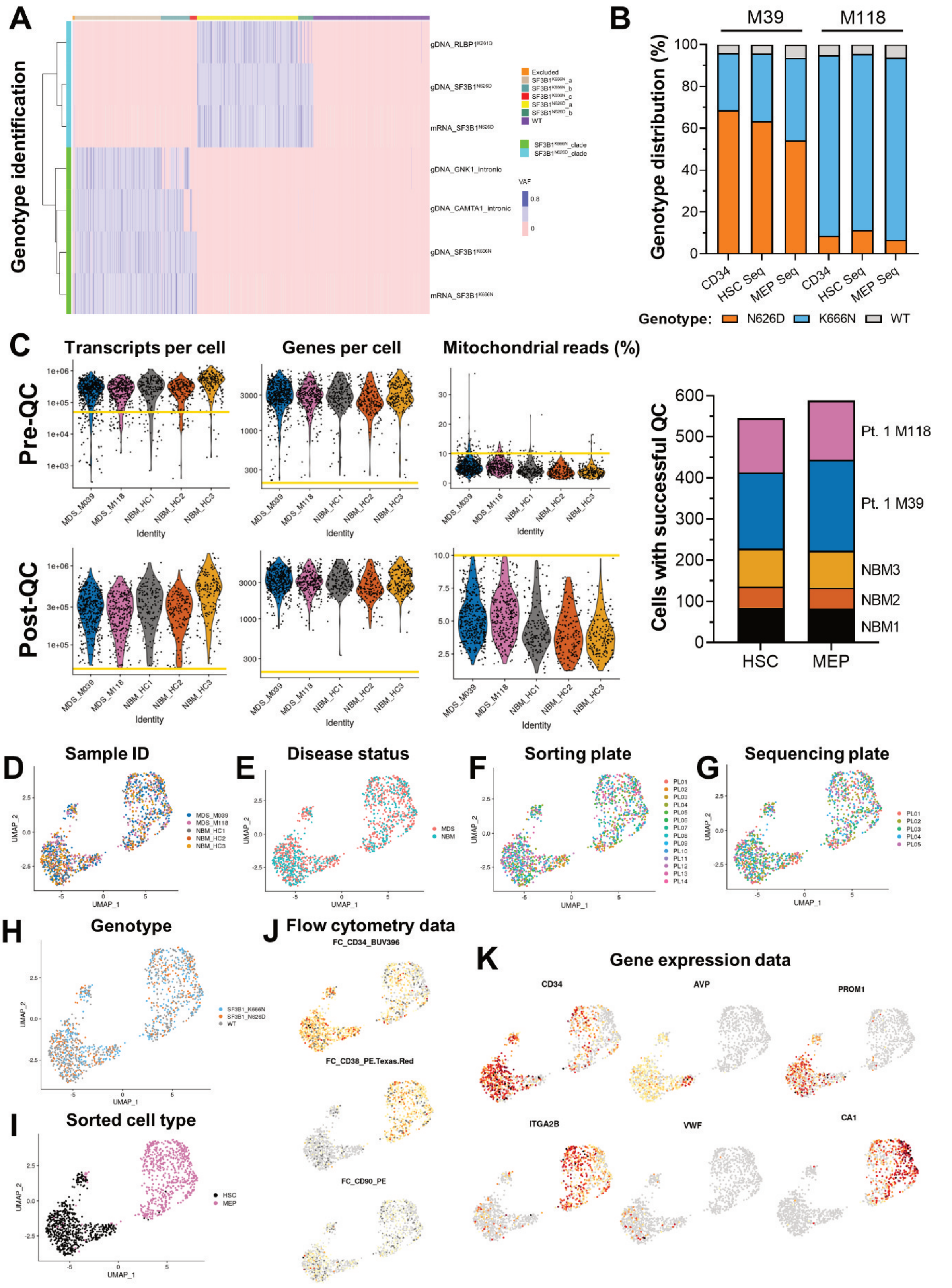


**Sup. Fig. 6) Quality control and baseline metrics of TARGET-seq genotype/transcriptional data**

## Sup. Fig. 6) Quality control and baseline metrics of TARGET-seq genotype/transcriptional data

**A)** Heatmap displaying detected VAFs in indicated gDNA and/or mRNA amplicons from individual cells with sufficient coverage analysed from Patient 1 (1615 cells, Month 39 post-diagnosis and Month 118 post-diagnosis) and 3 normal healthy donors (labelled HC throughout, 640 cells). Genotyping was based on a minimum read coverage of [(1.5 x blank well reads) + 30] of gDNA amplicons from *SF3B1* N626D and K666N mutations, as well as gDNA amplicons from clone-specific co-mutations (*RLBP1*$^{K261Q}$ [N626D], and *CAMTA1*$^{intronic}$ / *GNK1*$^{intronic}$ [K666N]). Cells were designated as wildtype if none of the 5 mutations were identified despite sufficient read coverage. Cells are arranged along the horizontal axis according to the genotyping calls based on which mutations were detected, including *SF3B1* K666N supported by two clade mutations (*SF3B1* K666N-a), *SF3B1* K666N supported by one clade mutation (*SF3B1* K666N-b), *SF3B1* K666N without detection of supporting clade mutations (*SF3B1* K666N-c), *SF3B1* N626D supported by the clade mutation (*SF3B1* N626D-a), *SF3B1* N6262D without detection of supporting clade mutation (*SF3B1* N626D-b), wildtype cells (WT) and excluded cells. VAFs are represented by color from red (0) to blue (max VAF, 0.8).

**B)** Patient 1 *SF3B1*$^{mt}$ frequency in sorted and genotyped TARGET-seq HSCs and MEPs as compared to back-calculated ddPCR-derived CD34$^{+}$ cell VAF estimates.

**C)** Violin plots displaying transcript counts per cell, obtained gene number per cell and mitochondrial read proportion per cell in TARGET-seq data, before (top row) and after (bottom row) quality control cut-offs (Min. 50,000 transcripts per cell; Min. 200 genes per cell; Max 10% mitochondrial reads per cell), and total count of cells successfully passing transcriptomic quality control.

**D-I)** UMAP of the total TARGET-seq dataset, separated by sample ID (**D**), disease status (**E**), sorting plate (**F**), sequencing plate (**G**), identified genotype (**H**) and FACS-purified cell type (**I**).

**J**/**K**) UMAP overlays displaying sorted flow cytometry index sorted cell fluorescence indices for CD34, CD38 and CD90 (**J**) and selected marker gene expression (**K**).

# Supplemental Figure 7

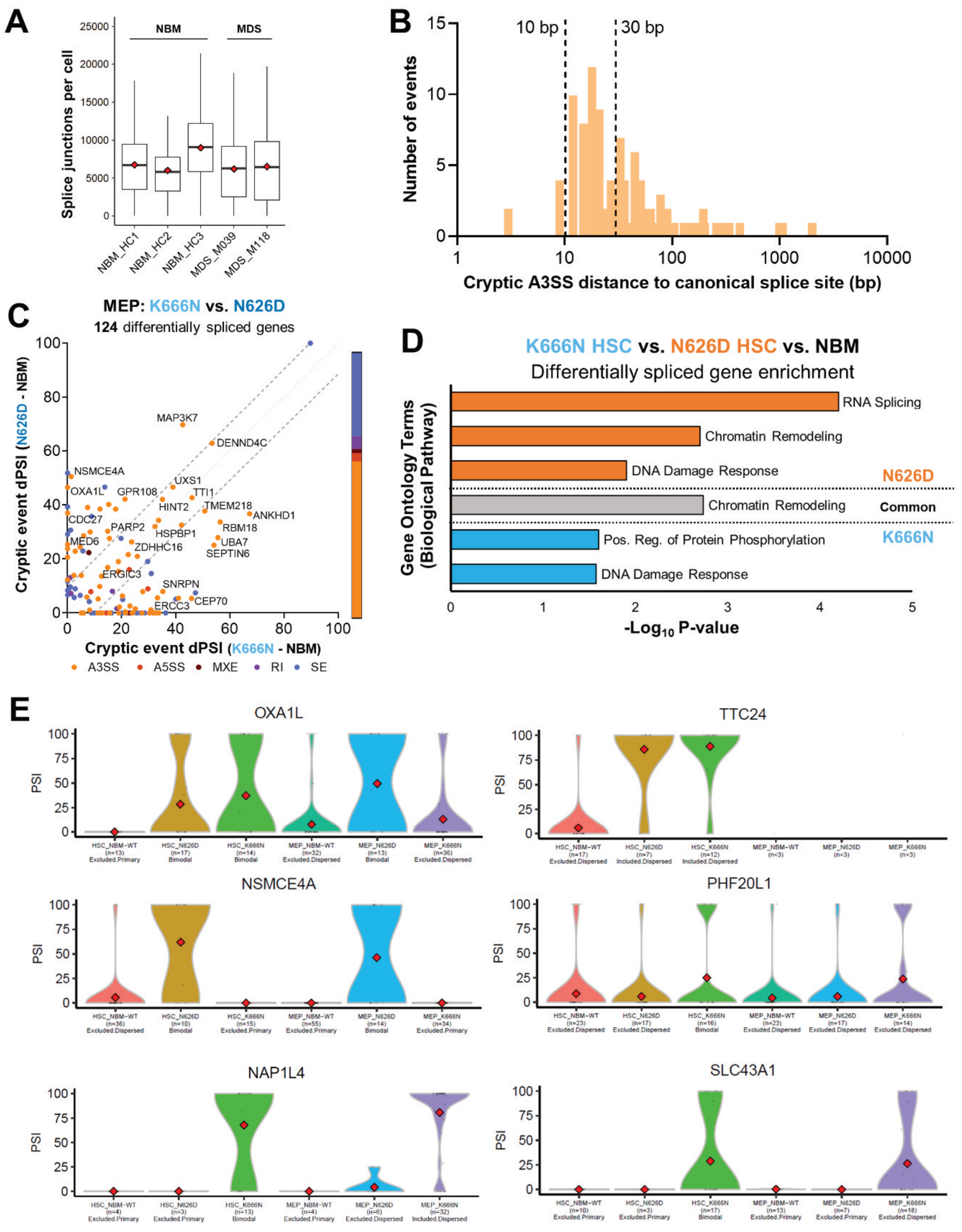


## Sup. Fig. 7) Alternative splicing analysis from single-cell TARGET-seq

**Sup. Fig. 7) Alternative splicing analysis from single-cell TARGET-seq**

**A)** Boxplot representation of the average number of SJs (splice junctions) detected per cell in each TARGET-seq sample. Boxes represent median and quartiles, and the central line represents the median for each group.

**B)** Histogram of absolute base pair (bp) distances from the detected cryptic A3SS sites to canonical splice sites. Lines at 10 and 30 bp demarcate the reported interval associated with *SF3B1* mis-splicing.[25]

**C)** Percent spliced in (PSI) difference values comparing *SF3B1*$^{K666N}$ and *SF3B1*$^{N626D}$ MEP PSIs against control PSIs (NBM, n = 3). Event types are annotated per color. SE = skipped exon, RI = retained intron, MXE = mutually exclusive exon usage, A5SS = alternative 5' splice site, A3SS = alternative 3' splice site.

**D)** Gene Ontology enrichment analysis of cryptic spliced genes in *SF3B1*$^{K666N}$ and *SF3B1*$^{N626D}$ HSC when compared to NBM HSCs, separated by predominant mutation association.
N626D: (N626D dPSI – K666N dPSI) > 10
Common: -10 < (N626D dPSI – K666N dPSI) < 10
K666N: (N626D dPSI – K666N dPSI) < -10

**E)** Violin plot of per-cell splicing event PSIs, separated by *SF3B1* genotype and cell type. Each point corresponds to one cell. Events include examples of global *SF3B1* mis-splicing (*OXA1L*, *TTC24*), *SF3B1*$^{N626D}$-specific mis-splicing (*NSMCE4A*) and *SF3B1*$^{K666N}$-specific mis-splicing (*PHF20L1*, *NAP1L4*, *SLC43A1*).

# References


1. Yokoyama, A., Kakiuchi, N., Yoshizato, T., Nannya, Y., Suzuki, H., Takeuchi, Y., Shiozawa, Y., Sato, Y., Aoki, K., Kim, S.K., et al. (2019). Age-related remodelling of oesophageal epithelia by mutated cancer drivers. Nature *565*, 312-317. 10.1038/s41586-018-0811-x.
2. Hoang, D.T., Vinh, L.S., Flouri, T., Stamatakis, A., von Haeseler, A., and Minh, B.Q. (2018). MPBoot: fast phylogenetic maximum parsimony tree inference and bootstrap approximation. BMC Evol Biol *18*, 11. 10.1186/s12862-018-1131-3.
3. Karcher, M.D., Palacios, J.A., Lan, S., and Minin, V.N. (2017). phylodyn: an R package for phylodynamic simulation and inference. Mol Ecol Resour *17*, 96-100. 10.1111/1755-0998.12630.
4. Schneider, V.A., Graves-Lindsay, T., Howe, K., Bouk, N., Chen, H.C., Kitts, P.A., Murphy, T.D., Pruitt, K.D., Thibaud-Nissen, F., Albracht, D., et al. (2017). Evaluation of GRCh38 and de novo haploid genome assemblies demonstrates the enduring quality of the reference assembly. Genome Res *27*, 849-864. 10.1101/gr.213611.116.
5. Melsted, P., Booeshaghi, A.S., Liu, L., Gao, F., Lu, L., Min, K.H.J., da Veiga Beltrame, E., Hjorleifsson, K.E., Gehring, J., and Pachter, L. (2021). Modular, efficient and constant-memory single-cell RNA-seq preprocessing. Nat Biotechnol *39*, 813-818. 10.1038/s41587-021-00870-2.
6. Stuart, T., Butler, A., Hoffman, P., Hafemeister, C., Papalexi, E., Mauck, W.M., 3rd, Hao, Y., Stoeckius, M., Smibert, P., and Satija, R. (2019). Comprehensive Integration of Single-Cell Data. Cell *177*, 1888-1902 e1821. 10.1016/j.cell.2019.05.031.
7. Oetjen, K.A., Lindblad, K.E., Goswami, M., Gui, G., Dagur, P.K., Lai, C., Dillon, L.W., McCoy, J.P., and Hourigan, C.S. (2018). Human bone marrow assessment by single-cell RNA sequencing, mass cytometry, and flow cytometry. JCI Insight *3*. 10.1172/jci.insight.124928.
8. Pellin, D., Loperfido, M., Baricordi, C., Wolock, S.L., Montepeloso, A., Weinberg, O.K., Biffi, A., Klein, A.M., and Biasco, L. (2019). A comprehensive single cell transcriptional landscape of human hematopoietic progenitors. Nat Commun *10*, 2395. 10.1038/s41467-019-10291-0.
9. McInnes, L., Healy, J., and Melville, J.J.a.e.-p. (2018). UMAP: Uniform Manifold Approximation and Projection for Dimension Reduction. arXiv:1802.03426.
10. Waltman, L., and van Eck, N.J. (2013). A smart local moving algorithm for large-scale modularity-based community detection. *86*, 471. 10.1140/epjb/e2013-40829-0.
11. Xie, Z., Bailey, A., Kuleshov, M.V., Clarke, D.J.B., Evangelista, J.E., Jenkins, S.L., Lachmann, A., Wojciechowicz, M.L., Kropiwnicki, E., Jagodnik, K.M., et al. (2021). Gene Set Knowledge Discovery with Enrichr. Curr Protoc *1*, e90. 10.1002/cpz1.90.
12. Rodriguez-Meira, A., O'Sullivan, J., Rahman, H., and Mead, A.J. (2020). TARGET-Seq: A Protocol for High-Sensitivity Single-Cell Mutational Analysis and Parallel RNA Sequencing. STAR Protoc *1*, 100125. 10.1016/j.xpro.2020.100125.
13. Li, H., and Durbin, R. (2010). Fast and accurate long-read alignment with Burrows-Wheeler transform. Bioinformatics *26*, 589-595. 10.1093/bioinformatics/btp698.
14. Dobin, A., Davis, C.A., Schlesinger, F., Drenkow, J., Zaleski, C., Jha, S., Batut, P., Chaisson, M., and Gingeras, T.R. (2013). STAR: ultrafast universal RNA-seq aligner. Bioinformatics *29*, 15-21. 10.1093/bioinformatics/bts635.
15. Li, H., Handsaker, B., Wysoker, A., Fennell, T., Ruan, J., Homer, N., Marth, G., Abecasis, G., Durbin, R., and Genome Project Data Processing, S. (2009). The Sequence Alignment/Map format and SAMtools. Bioinformatics *25*, 2078-2079. 10.1093/bioinformatics/btp352.
16. McKenna, A., Hanna, M., Banks, E., Sivachenko, A., Cibulskis, K., Kernytsky, A., Garimella, K., Altshuler, D., Gabriel, S., Daly, M., and DePristo, M.A. (2010). The Genome Analysis Toolkit: a MapReduce framework for analyzing next-generation DNA sequencing data. Genome Res *20*, 1297-1303. 10.1101/gr.107524.110.
17. Quinlan, A.R., and Hall, I.M. (2010). BEDTools: a flexible suite of utilities for comparing genomic features. Bioinformatics *26*, 841-842. 10.1093/bioinformatics/btq033.
18. Martin, M. (2011). Cutadapt Removes Adapter Sequences from High-Throughput Sequencing Reads. EMBnet Journal *17*, 10-12.
19. Liao, Y., Smyth, G.K., and Shi, W. (2014). featureCounts: an efficient general purpose program for assigning sequence reads to genomic features. Bioinformatics *30*, 923-930. 10.1093/bioinformatics/btt656.
20. Li, B., and Dewey, C.N. (2011). RSEM: accurate transcript quantification from RNA-Seq data with or without a reference genome. BMC Bioinformatics *12*, 323. 10.1186/1471-2105-12-323.
21. Shen, S., Park, J.W., Lu, Z.X., Lin, L., Henry, M.D., Wu, Y.N., Zhou, Q., and Xing, Y. (2014). rMATS: robust and flexible detection of differential alternative splicing from replicate RNA-Seq data. Proc Natl Acad Sci U S A *111*, E5593-5601. 10.1073/pnas.1419161111.
22. Garrido-Martin, D., Palumbo, E., Guigo, R., and Breschi, A. (2018). ggsashimi: Sashimi plot revised for browser- and annotation-independent splicing visualization. PLoS Comput Biol *14*, e1006360. 10.1371/journal.pcbi.1006360.
23. Wen, W.X., Mead, A.J., and Thongjuea, S. (2022). MARVEL: An integrated alternative splicing analysis platform for single-cell RNA sequencing data. bioRxiv, 2022.2008.2025.505258. 10.1101/2022.08.25.505258.
24. Mayakonda, A., Lin, D.C., Assenov, Y., Plass, C., and Koeffler, H.P. (2018). Maftools: efficient and comprehensive analysis of somatic variants in cancer. Genome Res *28*, 1747-1756. 10.1101/gr.239244.118.
25. Zhang, J., Ali, A.M., Lieu, Y.K., Liu, Z., Gao, J., Rabadan, R., Raza, A., Mukherjee, S., and Manley, J.L. (2019). Disease-Causing Mutations in SF3B1 Alter Splicing by Disrupting Interaction with SUGP1. Mol Cell *76*, 82-95 e87. 10.1016/j.molcel.2019.07.017.
